# Measuring Character Strengths and Promoting Positive Youth Development in Zambia: Initial Findings from the GROW Hopes for Life Study

**DOI:** 10.1007/s10566-024-09814-8

**Published:** 2024-08-01

**Authors:** Jonathan M. Tirrell, Mutale Sampa, Kit Wootten, Sion Kim Harris, Robert E. McGrath, Mataanana Mulavu, Ntazana Sindano, Lameck Kasanga, Oliver Mweemba, Dana McDaniel Seale, J. Paul Seale, Wilbroad Mutale

**Affiliations:** 1 Institute for Applied Research in Youth Development, Tufts University, Medford, USA; 2 University of Zambia, Lusaka, Zambia; 3 Augusta University, Augusta, USA; 4 Boston Children’s Hospital, Harvard Medical School, Boston, USA; 5 Fairleigh Dickinson University, Madison, USA; 6 University of North Carolina, Charlotte, USA; 7 Serenity Harm Reduction Programme Zambia, Lusaka, Zambia; 8 Global Resilience Oral Workshops, Lusaka, Zambia

**Keywords:** Character strengths, Positive youth development, Zambia, Unhealthy alcohol use, GROW Hopes for Life study

## Abstract

**Background:**

The Global Resilience Oral Workshops (GROW) Free and Strong programs take a strengths-based, positive youth development (PYD) approach to promoting thriving. Through both prevention (GROW Strong) and intervention (GROW Free) exercises, these programs aim to build character and emotional resilience while also lowering unhealthy alcohol use.

**Objective:**

To meaningfully assess the impact of the GROW programs on health and PYD, ecologically and psychometrically valid measures of character strengths were needed, with a focus on the strengths of hope, forgiveness, spirituality, prudence, and self-control (self-regulation) promoted by GROW.

**Method:**

We tested a series of exploratory and confirmatory factor analyses of these five key constructs using two samples: a school-based youth sample enrolled in GROW Strong (*n* = 460; *M*_*age*_ = 15.04 years, *SD*_*age*_ = 1.21; 53.0% female); and a community-based adult sample enrolled in GROW Free (*n* = 457; *M*_*age*_ = 20.60 years, *SD*_*age*_ = 1.88; 49.7% female); both enrolled using a waitlist-control design.

**Results:**

Measures demonstrated strong invariance across specific subgroups present in the data sets, with differences emerging across ages, urban/rural locations, and baseline study conditions.

**Conclusions:**

To meaningfully document PYD programs and character development in the majority world, measurement models must be theory-predicated, robust, and empirically validated for the specific context. The results provide evidence for such a measure that will be useful in future intervention studies promoting character strengths to address unhealthy alcohol use in Zambia.

**Supplementary information:**

The online version contains supplementary material available at 10.1007/s10566-024-09814-8.

In recent decades, research and practice have increasingly taken a strengths-based, positive youth development (PYD) approach to promoting thriving among the world’s youth (Koller & Verma, 2017; Lerner et al., 2021a, 2021b; Lerner et al., 2019, 2021a, 2021b; YouthPower Learning, 2017). Earlier deficits-based perspectives considered youth as a time of problems or risks to be managed (Roth & Brooks-Gunn, 2003); and adolescence as a time of crisis (Erickson, 1968), developmental disturbance (Freud, 1969), disruption (Soto & Tackett, 2015; see also Bates & McGrath, 2024), and “storm and stress” (Hall, 1904). In contrast, PYD considers youth as assets to be developed and as contributing producers of their own development, engaging in mutually influential relations with their contexts (Lerner, 2021; Overton, 2015). Such a shift in the philosophy and practice of youth programs also requires a shift in their measurement and evaluation.

As noted by UNICEF (see YouthPower Learning, 2017), PYD programs are being adopted globally; however, particularly in the majority world (sometimes referred to as the Global South or low- and middle-income countries), there remains a lack of strong theoretical underpinnings for such programs, of robust and consistent measurement of program processes and outcomes, and of rigorous longitudinal studies assessing program impact. Evidence is needed to describe, explain, and optimize (see Baltes et al., 1977) PYD in the majority world. To understand and promote the efficacy of strengths-based approaches, evidence is further needed to understand whether and how promoting strengths might also promote prevention of, and/or recovery from, health-related risk behaviors among youth. As well, foundations and organizations are increasingly interested in such evidence. For example, the Templeton World Charity Foundation, in its Global Innovations in Character Development initiative, seeks to understand the role of character strengths in promoting health-related outcomes (Templeton World Charity Foundation, n.d).

In the present study we aim to take an initial step toward responding to the above-noted calls from UNICEF and Templeton World Charity Foundation. As part of the broader Global Resilience Oral Workshops (GROW) *Hopes for Life* study, known in Zambia as “Ziyembekezo Za Umoyo,” we present steps taken to establish a theory-predicated, ecologically valid, and psychometrically strong measurement model for future evaluations of the GROW “Hopes for Life” programs. The curriculum includes two strengths-based PYD programs in Zambia: GROW Strong, a resilience-training prevention program; and GROW Free, a prevention and recovery program for unhealthy alcohol use (see Harris et al., 2020; Seale, 2014; Seale et al., 2021). Both GROW Strong and GROW Free are faith-based PYD programs that seek to address unhealthy alcohol use, a growing health risk among youth in Zambia (see Mungandi et al., 2022; World Health Organization, 2018). These two programs take a strengths-based, PYD approach to fostering resilience and recovery from unhealthy alcohol use by promoting five character strengths found to be correlated with lower alcohol use, specifically, hope (e.g., Baines et al., 2016), forgiveness (e.g., Webb et al., 2017), spirituality (e.g., Tumwesigye et al., 2013), prudence (e.g., Kabakci, 2019), and self-control (e.g., Logan et al., 2010). The goal of this study is to establish a useful measurement model for assessing these strengths.

## Unhealthy Alcohol Use and Youth in Zambia

As emphasized in the United Nations (2015, n.d.) Sustainable Development Goals 2030 agenda, strengthening the prevention and treatment of substance use disorders is needed (see also Patel et al., 2018). In particular, unhealthy alcohol use is a serious issue affecting youth worldwide. Unhealthy alcohol use involves a spectrum of alcohol use that ranges from at-risk use (exceeding recommended low-risk limits) to problematic use and alcohol use disorder (Patel & Balasanova, 2021).

Alcohol remains the leading global risk for disease and premature death among people ages 15 to 49 years (Flor & Gakidou, 2020), at least since 1990 (Global Burden of Disease, 2018). It is the third leading cause of premature death and disability in the world, causing three million deaths per year, and contributing to more than 200 disease and injury conditions (World Health Organization, 2022). Low- and middle-income countries have been particularly impacted. In 2018, the *Lancet* Commission on global mental health and sustainable development set off an alarm, calling the international community to address the profound lack of mental health and substance use treatment services in the majority world (Patel et al., 2018).

Adolescence is a critical time to address the unhealthy use of alcohol. Alcohol use in adolescence is associated with future alcohol-related problems (DeWit et al., 2000; Grant & Dawson, 1997; Grant et al., 2001); with sexual risk-taking (Stueve & O’Donnell, 2005); and with academic problems and delinquent behavior (Ellickson et al., 2003). Risks to healthy brain development include impairment in the domains of attention, information processing, memory, and executive functioning (Pfefferbaum et al., 2017; Squeglia et al., 2015).

Over one-third of Zambian secondary school students reported initiating alcohol use (other than a few sips) at age 13 or younger (Birhanu et al., 2014; Swahn et al., 2011). Despite the minimum legal drinking age of 18 in Zambia, nearly half of Zambian adolescents ages 13–16 reported past-month alcohol use, and their early alcohol use was associated with hazardous and harmful drinking patterns; two-thirds of Zambian 15- to 19-year-olds using alcohol reported “binge” drinking (World Health Organization, 2018; see also Swahn et al., 2011). Among adolescents and young adults in Zambia and, specifically the capital city of Lusaka, alcohol consumption has been noted as very high. Contributors to use include peer influence, unemployment, experimentation, boredom, rebellion, other challenges associated with poverty, easy access to cheap alcohol, parental drinking habits, and lack of parental guidance (Mungandi et al., 2022).

Taken together, unhealthy alcohol use represents a major risk for youth in general and more specifically in Zambia. A key question of the broader GROW *Hopes for Life* study, therefore, is whether taking a PYD approach, by promoting youth character strengths, is effective in preventing or reducing such risks as unhealthy alcohol use.

## Taking a PYD Approach: Addressing Unhealthy Alcohol use by Promoting Character Strengths

Although adolescent years may bring curiosity, experimentation, and impulsiveness in relation to alcohol use (Casey et al., 2008; Stautz & Cooper, 2013), evidence exists that certain character strengths may serve as protective factors against unhealthy alcohol use. Indeed, character strengths are associated with positive outcomes among adolescents, including greater wellbeing (Duan et al., 2014; Froh et al., 2008; Toner et al., 2012), greater positive emotion (Martinez-Marti & Ruch, 2014), greater life satisfaction (Peterson et al., 2007), and greater self-esteem (Wood et al., 2011).

Specific to alcohol use, the virtue of temperance, which is associated with such character strengths as forgiveness, humility, prudence, and self-control, has been associated with abstinence, lower-risk drinking, and fewer consequences among at-risk drinkers (Logan et al., 2010). Lower-risk drinking and decreased alcohol use have also been correlated with prudence (Kabakci, 2019), forgiveness (Knight et al., 2007; Webb et al., 2017), and hope (Baines et al., 2016; Ferrari et al., 2012; Mathis et al., 2009). Religiosity and spirituality are also associated with lower levels of alcohol and drug use (Knight et al., 2007; Miller et al., 2000; see also Tumwesigye et al., 2013). Spirituality is perhaps especially important during adolescent development (Roehlkepartain et al., 2006). During adolescence, spirituality is highly protective for adolescents against alcohol problems. Miller et al. (2000) found that adolescents with a strong personal relationship with God or a higher power were 70–80% less likely to engage in heavy substance use or substance abuse. Spirituality is theorized to increase resilience in adolescence through the development of secure attachment relationships, the creation of additional sources of social support, guiding conduct and moral values, and providing opportunities for personal growth and development by exposing youth to a wide range of character strengths (Crawford et al., 2006).

## The Present Study

In the present study, we aimed to establish measurement tools useful for assessing character strengths and PYD in Zambia. We report findings from developing ecologically valid and psychometrically strong measures of character strengths to use in subsequent evaluation studies of two curricula being implemented in the ongoing GROW Hopes for Life clinical trial through a Schools program and a Communities program. Both curricula are faith-based PYD programs designed to address unhealthy alcohol use in Zambia: GROW Strong and GROW Free (see Harris, 2020; Seale et al., 2021; see also Seale, 2014). Both programs focus on the character strengths of hope, forgiveness, spirituality, prudence, and self-control to promote PYD and prevention of, or recovery from, unhealthy alcohol use.

Specifically, the Hopes for Life – Schools program takes a preventive, resilience-training approach with 13- to 17-year-olds by first teaching GROW Strong, then teaching GROW Free. This approach is taken because the GROW Strong program aims to promote resilience, and closing with the GROW Free program is intended to raise awareness about the risks of unhealthy alcohol use and prevent youth from engaging in it. In turn, the Hopes for Life – Communities program is an intervention program for those ages 18 to 24 experiencing unhealthy alcohol use and, therefore, first teaches GROW Free, as it is focused on recovery. By closing with the GROW Strong program, those recovering are intended to be equipped with the skills and resilience to maintain recovery.

Accordingly, to assess the impact and effectiveness of GROW Hopes for Life on promoting character strengths and PYD, a meaningful measurement model with strong psychometric properties was needed. We therefore used baseline data collected from two distinct samples—a school-based sample of younger youth participating in the GROW Hopes for Life – Schools program; and a community-based sample of older youth with unhealthy alcohol use participating in the Hopes for Life – Communities program—to establish a psychometrically strong measurement model. Establishing ecologically and psychometrically valid measure for assessing PYD and evaluating PYD programs in majority world contexts would therefore contribute to the above-noted needs in the field (e.g., Templeton World Charity Foundation, n.d.; YouthPower Learning, 2017). Furthermore, validating the measurement model prior to the implementation of the interventions (i.e., at baseline) in the present study will enhance the validity of the results of subsequent evaluations of the interventions.

## Method

### Participants

The present study involved two distinct samples: a school-based sample ages 13 to 17 years (*n* = 460; *M*_*age*_ = 15.04 years, *SD*_*age*_ = 1.21; 53.0% female); and a community-based sample ages 18 to 24 years (*n* = 457; *M*_*age*_ = 20.60 years, *SD*_*age*_ = 1.88; 49.7% female). The school and community samples were tested separately to validate distinct measurement models for the respective programs, given their respective foci (resilience and recovery) and developmental ages, as described above.

### Ethics Review Process

Researchers obtained ethics approval from the University of Zambia Biomedical Research Ethics Committee, a local Research Ethics Committee (REC), and the National Health Research Authority (NHRA), which serves as the regulatory body responsible for overseeing research in Zambia. Ethics approval was also obtained by Augusta University and Boston Children's Hospital Institutional Review Boards. The research team fully adhered to the Institutional Review Board regulations in Zambia.

Informed consent was obtained from all participants prior to their participation in the study. For minors below the age of 18, youth assent and parental consent were required. All participants were informed that their involvement in the study was entirely voluntary and that they would not face any adverse consequences if they chose not to participate or declined to answer any questions. The research team upheld strict confidentiality measures and de-identified the data for analysis to ensure the privacy and anonymity of participants.

### Measures

Measures of five constructs were examined: hope, forgiveness, spirituality, prudence, and self-control. The research team compiled items from several measures and also generated new items based on the Zambian context and/or the goals of the programs. The measures were translated from English to the local language, Nyanja, and reviewed by the Zambian-based research team for accuracy and clarity. The measures relied on youth’s self-reported perceptions and assessments of how much they relate to or identify with the items. All participating youth were given the scales in the same order. For all measures, response options ranged from “0” (*Completely disagree*) to “10” (*Completely agree*), with higher scores reflecting higher self-ratings on the construct. Supplementary Table 1 presents items for all measures used.

#### Hope

To assess hope, we compiled a total of 18 items: four items from the VIA Inventory of Strengths (VIA-IS; Park & Petersen, 2006); 12 items from the 4-H Study of PYD (see Schmid et al., 2011); and two items generated by the research team. Regarding internal consistency reliability for the full item pool, McDonald’s omega was 0.853 for the school-based sample and 0.905 for the community-based sample.

#### Forgiveness

To assess forgiveness, we used 10 items: one item from the VIA-IS (Park & Petersen, 2006); three items from the Brief Multidimensional Measure of Religiousness/Spirituality (BMMRS; Masters, 2020); three items derived from the Compassion International Study of PYD (e.g., Tirrell et al., 2019); and three items generated by the research team. McDonald’s omega was 0.819 for the school-based sample and 0.890 for the community-based sample.

#### Spirituality

We used eight items to assess spirituality: five items related to a factor of transcendence from the Measure of Diverse Adolescent Spirituality (MDAS; King et al., 2017; see also Tirrell et al., 2019); and three items from the VIA-IS (Parker & Petersen, 2006) Spirituality scale. McDonald’s omega was 0.675 for the school-based sample and 0.944 for the community-based sample.

#### Prudence

To assess prudence, we used seven items: four items from the VIA-IS (Park & Petersen, 2006); and three items generated by the research team. McDonald’s omega was 0.771 for the school-based sample and 0.869 for the community-based sample.

#### Self-Control (Self-Regulation)

For self-control, we used seven items: four items from the VIA-IS (Park & Petersen, 2006); and three items generated by the research team. McDonald’s omega was 0.737 for the school-based sample and 0.839 for the community-based sample.

### Procedure

Data collection was overseen by professionals affiliated with the University of Zambia and the Serenity Harm Reduction Programme Zambia (SHARPZ). Lusaka and Sinda districts were chosen as the study locations because they provided a representation of both urban (Lusaka) and rural (Sinda) contexts, and because of existing community contacts through previous SHARPZ and GROW projects. To prepare for data collection, SHARPZ conducted a mapping exercise in Lusaka and Sinda. After identifying potential study locations therein, a randomization process was implemented, resulting in the selection of four distinct operational regions within each district. Subsequently, staff members from the SHARPZ team conducted a sensitization campaign at the selected study locations to raise awareness about the project. Each location included a combination of four schools and four community sites.

Before data collection, the team recruited research assistants who completed a four-day training on the study objectives, procedures, tools, and research ethics. During the training of research assistants for data collection, the study tools were tested among both school and community members in Lusaka. After receiving feedback from the research assistants (e.g., regarding survey comprehension, wording, response scales), revisions were made to the survey.

Enrolment of students in schools depended on meeting two specific criteria: falling within the age range of 13 to 17 years old and presently attending the 8th grade. To assess individuals within the community and determine their suitability for participation in the study, eligibility criteria included: being between the ages of 18 to 24, being able to read English or Nyanja, having known or suspected unhealthy alcohol use, and demonstrating an alcohol risk score ≥ 2 on the Alcohol, Smoking and Substance Involvement Screening Test (ASSIST; Humeniuk et al., 2008) collected at baseline. Once informed consent was obtained, participants were randomized to either a treatment group or a waitlist-control group. Baseline data were collected from both student and community participants across conditions. The research team conducted individual interviews with the participants to complete the survey using tablets. Sensitive questions related to substance use and sexual risk factors were administered as audio-assisted items using headphones to enhance privacy and confidentiality. Surveys took approximately one hour to complete.

### Data Analysis

The present research addressed the overarching goal of creating an ecologically and psychometrically valid measurement model to assess five character strengths promoted by the GROW Strong and GROW Free programs in Zambia: hope, forgiveness, spirituality, prudence, and self-control. Upon establishing measures, we conducted exploratory analyses regarding the relations among the latent constructs. Accordingly, data analysis involved multiple steps.

Data from the school and community samples were analyzed separately to validate distinct measurement models, given the distinct target ages (adolescents and young adults) and purposes (prevention and intervention, respectively) of the programs. Analyses were conducted using Mplus Version 8 (Muthén & Muthén, 1998–2017). We used multiple goodness of fit indices as recommended by Brown (2006). Absolute fit was tested by assessing the standardized root mean square residual (SRMR), with values closer to 0 indicating better fit (Brown, 2006) (also due to an oversensitivity of the χ^2^ difference test to small differences in large samples; see Rutkowski & Svetina, 2017). Parsimony–corrected fit was assessed by evaluating the root mean square error of approximation (RMSEA) and its confidence interval, with values closer to 0 indicating better model fit (Brown, 2006). The suggested upper bounds, or cut-off values, of acceptable fit for the SRMR and RMSEA are 0.08, and ideally less than 0.05 (Brown & Cudeck, 1993; Hu & Bentler, 1999; Stieger, 1990). Comparative fit, the evaluation of the specified solution in comparison to a null model in which no items are correlated, was tested with the comparative fit index (CFI) and the Tucker-Lewis Index (TLI), with values closer to 1 indicating better model fit (Brown, 2006). The suggested lower bounds, or cut-off values, of acceptable fit for the CFI and TLI are 0.90, and ideally above 0.95 (Bentler, 1990).

For the school- and community-based samples, respectively, we first analyzed each specific construct’s item pool independently using exploratory factor analysis (EFA) to test its purported factor structure. Geomin rotation (Mplus default) was used for a parsimonious factor pattern matrix. A combination of criteria was used to determine the adequate number of factors to retain, including a scree plot (Cattell, 1966), parallel analysis (Glorfeld, 1995; Horn, 1965), and the above-noted multiple goodness of fit indices as recommended by Brown (2006). To determine which items to retain within factors, we followed the suggested cut-off values from Comrey and Lee (1992)—0.32 poor, 0.45 fair, 0.55 good, 0.63 very good, and 0.71 excellent—retaining items with loadings that were good to excellent for a strong and parsimonious model. Across the iterative EFAs, items were removed for low factor loadings or cross-loadings with other factors until acceptable model fit was achieved.

For the prudence and self-control items, the research team decided to combine the item pools to test the EFA, given the strong conceptual overlap and wording similarity across the items. The remaining three item pools (for hope, spirituality, forgiveness, respectively) were first tested independently across the two samples. Iterative EFAs were tested for each construct until a good-fitting model was demonstrated. Then a confirmatory factor analysis (CFA) was tested on the full measurement model (i.e., with all refined factors in one model) for each sample. The full-model CFAs were then further refined, if and as needed, until acceptable model fit was achieved.

We then tested the final measurement models for between-group measurement invariance to assess the robustness of the measurement model and to determine whether it was appropriate and meaningful to compare groups present within the data set on these measures (see Little, 1997, 2013). We tested demographic subgroups present within the data set, namely, gender (male and female), age (younger [13–14 years for the school-based sample and 18–20 for the community-based sample] and older [15–17 for the school-based sample and 21–24 for the community-based sample]), living location (urban and rural), and study group condition assignment (intervention and waitlist control).

Between-group measurement invariance was tested using a series of CFAs with added constraints, described further below (see Little, 2013). For all models, maximum likelihood estimation with robust standard errors was used. We used the criterion suggested by Cheung and Rensvold (2002) and further supported by King et al. (2017) for establishing invariance if changes in CFI are less than or equal to 0.01 across subsequent models.

Between-group measurement invariance refers to the comparability of measures across specific groups (i.e., whether measures assess the same latent constructs and demonstrate strong psychometric properties). For between-group measurement invariance, we first tested for configural invariance (i.e., equivalence of the measurement model structure) across groups. Next, we tested for metric (weak factorial) invariance (i.e., equivalence of the factor loadings across subgroups) by equating the factor loadings across groups and allowing the latent variances in one group to be freely estimated. Then, we tested for scalar (strong factorial) invariance (i.e., equivalence of the factor loadings *and* item intercepts across subgroups) by equating the factor loadings and item intercepts across groups and allowing the latent variances and latent means in one group to be freely estimated (see Little, 1997, 2013). Establishing invariance then enabled us to compare the latent means of the constructs across the respective groups.

## Results

In this sample, 0.0% of the item-level data were missing. No missing data procedures were therefore needed. We first summarize the final measurement model that resulted from the iterative series of EFAs within each construct and each sample (as it is beyond the scope of the present article to report the full results of each series of EFAs tested across the item pools for each respective construct across the two samples). We then present the results for the between-group measurement invariance tests for each sample.

### Preliminary Tests Establishing the Measurement Models

For the school-based sample, the preliminary EFAs resulted in a model including 16 of the original 50 items (see Supplementary Table 2), representing four distinct factors with good to excellent factor loadings. From the hope items emerged two distinct and good-fitting factors: “hope through hard times” (three items) and “hopeful future expectations” (five items). The prudence and self-control items did not form distinct factors in the school-based sample; instead, a single, four-item factor emerged. The forgiveness items formed a four-item factor. Last, no good-fitting model or factor could be derived from the spirituality items, likely due to a ceiling effect and lack of variance in item responses; model fit was poor and factor loadings were low. Together, a CFA tested on the final model of four factors and 16 items tested on the school-based sample demonstrated good fit to the data: SRMR = 0.046; RMSEA = 0.033 [0.021, 0.044]; CFI = 0.954; TLI = 0.941. Correlations among the latent factors ranged from *r* = 0.253 (forgiveness with hopeful future expectations) to *r* = 0.677 (hope through hard times with hopeful future expectations), indicating that the four latent factors were distinct (i.e., no correlations were above 0.8) and positively manifolded in the present sample (see Table 1).

**Table 1:** Correlations among the latent factors established with the school-based sample

	1. Hope in hardship	2. Hopeful future expectations	3. Forgiveness	5. Prudence/ Self-control
1. Hope in hardship	–			
2. Hopeful future expectations	.677**	–		
3. Forgiveness	.263*	.253**	–	
5. Prudence/Self-control	.637**	.633**	.489**	–

The final model tested on the school-based sample demonstrated good fit to the data: χ^2^ (95) = 142.752, *p* = .0011; RMSEA = .033 [.021, .044]; CFI = .954; TLI = .941; SRMR = .046. * *p* < .01. ** *p* < .01

For the community-based young adult sample, the preliminary EFAs resulted in a model including 44 of the original 50 items (see Supplementary Table 3). The 44 items reflected six factors: hope through hard times (six items); hopeful future expectations (10 items); forgiveness (eight items); spirituality (eight items); prudence (six items); and self-control (six items). This final model CFA tested on the community-based sample demonstrated good fit to the data: SRMR = 0.045; RMSEA = 0.031 [0.027, 0.035]; CFI = 0.953; TLI = 0.949. Correlations among the latent factors ranged from *r* = 0.544 (hope through hard times with spirituality) to *r* = 0.842 (prudence with self-control) (see Table 2). It should be noted that, although a model may demonstrate good fit in regard to its fit statistics, it may still be the case that the correlations among the latent factors might be high and, thus, interpretation should be made with caution, as high correlations among latent factors suggest there may be no distinction among them. Here, although a good-fitting model was established with prudence and self-control modeled as separate factors, the high correlation between them (i.e., above 0.8; *r* = 0.842) suggests that the factors may not be distinct in the community-based sample. The remaining correlations suggest that the factors are distinct and positively manifolded.

**Table 2:** Correlations among the latent factors established with the community-based sample

	1. Hope in hardship	2. Hopeful future expectations	3. Forgiveness	4. Spirituality	5. Prudence	6. Self-control
1. Hope in hardship	–					
2. Hopeful future expectations	.696**	–				
3. Forgiveness	.586**	.553**	–			
4. Spirituality	.544**	.575**	.620**	–		
5. Prudence	.654**	.748**	.632**	.588**	–	
6. Self-control	.595**	.630**	.739**	.594**	.842**	–

The final model tested on the community-based sample demonstrated good fit to the data: χ^2^ (873) = 1250.958, *p* = .0000; RMSEA = .031 [.027, .035]; CFI = .953; TLI = .949; SRMR = .045. ** *p* < .001

### Testing Between-Group Measurement Invariance

With parsimonious and robust models of character strengths established for the school- and community-based samples, respectively, we next tested for between-group measurement invariance across gender, across two age groups within each sample, across urban/rural districts, and across baseline study condition assignments (we note that, although these data are baseline data, collected prior to implementation of the intervention programs, we tested for invariance across study condition assignments to justify the use of these measures in future post-intervention evaluations). Table 3 presents all results for the school-based sample; Table 4 presents all results for the community-based sample.

**Table 3:** Model fit statistics for invariance tests of a 4-factor model of character strength for the school-based sample

Between-group invariance tests by gender (male and female)							
Model	χ^2^ (df)	*p*	RMSEA [90% CI]	SRMR	CFI	TLI	Pass? [ΔCFI ≤ .01]
1. Configural	264.371 (190)	.0003	.041 [.029, .053]	.057	.933	.915	
2. Weak/Loading	265.690 (202)	.0018	.037 [.023, .049]	.066	.943	.932	Yes [ΔCFI = .010]
3. Strong/Intercept	284.244 (214)	.0009	.038 [.025, .049]	.071	.937	.929	Yes [ΔCFI = .006]
Between-group invariance tests by age (younger and older; 13–14 and 15–18 years)							
Model	χ^2^ (df)	*p*	RMSEA [90% CI]	SRMR	CFI	TLI	Pass? [ΔCFI ≤ .01]
1. Configural	289.942 (190)	.0000	.048 [.036, .059]	.059	.912	.889	
2. Weak/Loading	302.468 (202)	.0000	.048 [.035, .057]	.076	.911	.895	Yes [ΔCFI = .001]
3. Strong/Intercept	317.155 (214)	.0000	.046 [.035, .056]	.081	.909	.898	Yes [ΔCFI = .002]
Between-group invariance tests by district – Lusaka (urban) and Sinda (rural)							
Model	χ^2^ (df)	*p*	RMSEA [90% CI]	SRMR	CFI	TLI	Pass? [ΔCFI ≤ .01]
1. Configural	240.591 (190)	.0076	.038 [.020, .052]	.059	.952	.940	
2. Weak/Loading	273.433 (202)	.0006	.043 [.029, .056]	.090	.933	.920	No [ΔCFI = .019]
3. Strong/Intercept	306.959 (213)	.0000	.048 [.035, .060]	.101	.912	.902	No [ΔCFI = .021]
Between-group invariance tests by condition (waitlist control and intervention)							
Model	χ^2^ (df)	*p*	RMSEA [90% CI]	SRMR	CFI	TLI	Pass? [ΔCFI ≤ .01]
1. Configural	267.122 (190)	.0002	.042 [.029, .053]	.056	.933	.915	
2. Weak/Loading	266.904 (202)	.0015	.037 [.024, .049]	.064	.943	.933	Yes [ΔCFI = .010]
3. Strong/Intercept	285.399 (214)	.0008	.038 [.025, .049]	.068	.938	.930	Yes [ΔCFI = .005]

χ^2^ = chi-square value. df = degrees of freedom. *p* = p-value. RMSEA = root mean error of approximation. CI = confidence interval. SRMR = standardized root mean square residual. CFI = comparative fit index. TLI = Tucker-Lewis index. ΔCFI = change in CFI value

**Table 4:** Model fit statistics for invariance tests of a 6-factor model of character strength for the community-based sample

Between-group invariance tests by gender (male and female)							
Model	χ^2^ (df)	*p*	RMSEA [90% CI]	SRMR	CFI	TLI	Pass? [ΔCFI ≤ .01]
1. Configural	2573.895 (1746)	.0000	.046 [.042, .050]	.057	.910	.903	
2. Weak/Loading	2587.688 (1784)	.0000	.045 [.041, .048]	.062	.913	.908	Yes [ΔCFI = .003]
3. Strong/Intercept	2631.262 (1822)	.0000	.044 [.041, .048]	.063	.913	.909	Yes [ΔCFI = .000]
Between-group invariance tests by age (younger and older; 17–20 and 21–25 years)							
Model	χ^2^ (df)	*p*	RMSEA [90% CI]	SRMR	CFI	TLI	Pass? [ΔCFI ≤ .01]
1. Configural	2592.758 (1746)	.0000	.046 [.043, .050]	.058	.909	.901	
2. Weak/Loading	2619.620 (1784)	.0000	.046 [.042, .049]	.064	.910	.904	Yes [ΔCFI = .001]
3. Strong/Intercept	2657.372 (1822)	.0000	.045 [.041, .049]	.064	.910	.907	Yes [ΔCFI = .000]
Between-group invariance tests by district – Lusaka (urban) and Sinda (rural)							
Model	χ^2^ (df)	*p*	RMSEA [90% CI]	SRMR	CFI	TLI	Pass? [ΔCFI ≤ .01]
1. Configural	2600.050 (1746)	.0000	.047 [.043, .050]	.058	.901	.893	
2. Weak/Loading	2634.704 (1784)	.0000	.046 [.042, .050]	.073	.901	.895	Yes [ΔCFI = .000]
3. Strong/Intercept	2715.355 (1822)	.0000	.047 [.043, .050]	.075	.896	.892	Yes [ΔCFI = .005]
Between-group invariance tests by condition (waitlist control and intervention)							
Model	χ^2^ (df)	*p*	RMSEA [90% CI]	SRMR	CFI	TLI	Pass? [ΔCFI ≤ .01]
1. Configural	2567.336 (1746)	.0000	.046 [.042, .049]	.059	.914	.907	
2. Weak/Loading	2587.812 (1784)	.0000	.045 [.041, .048]	.063	.916	.911	Yes [ΔCFI = .002]
3. Strong/Intercept	2640.683 (1822)	.0000	.045 [.041, .048]	.064	.914	.911	Yes [ΔCFI = .002]

χ^2^ = chi-square value. df = degrees of freedom. *p* = p-value. RMSEA = root mean error of approximation. CI = confidence interval. SRMR = standardized root mean square residual. CFI = comparative fit index. TLI = Tucker-Lewis index. ΔCFI = change in CFI value

In the school-based sample (see Table 3), strong invariance was established between boys and girls, justifying comparisons to be made across gender using this measurement model. No significant differences emerged between boys and girls in regard to the means of the latent factors in the school-based sample. Strong invariance was also established across the younger and older school-based groups, and there were no significant differences in regard to the latent means. However, grouping the data by these age bins in the school-based sample negatively affected model fit as indicated by the TLI dropping below 0.9; accordingly, the model might need further refining if age-based conclusions are intended to be drawn from use of these measures. For urban and rural location, although the configural model was found to be equivalent across the school-based groups, neither weak (loading; metric) nor strong (intercept; scalar) invariance was demonstrated, suggesting that these measures are not invariant between samples from urban and rural contexts and thus such context-specific comparisons should be made with caution. Last, strong invariance was established across study condition. In comparing means of the latent constructs across study conditions in the school-based sample, at baseline, youth assigned to the intervention condition reported significantly lower levels of hope through hardship (-0.212 standardized mean difference [SMD], *p* = 0.041) and of prudence/self-control (-0.420 SMD, *p* < 0.001) compared to youth assigned to the waitlist control group.

In the community-based sample (see Table 4), strong invariance was established across gender, with no significant differences in latent means emerging. Strong invariance was also established across age groups in the community-based sample; regarding latent means, older community youth reported significantly lower levels of hopeful future expectations (-0.219 SMD, *p* = 0.008), and significantly lower levels of spirituality (-0.215 SMD, *p* = 0.002), compared to younger community youth. Although strong invariance was also established across urban and rural locations in the community-based sample, the model fit fell below acceptable levels as indicated by TLI below 0.9. Rural community youth (from Sinda) reported significantly lower levels of all six character strengths: hope through hardship (-0.407 SMD, *p* < 0.001); hopeful future expectations (-0.522 SMD, *p* < 0.001); prudence (-0.423 SMD, *p* < 0.001); self-control (-0.199 SMD, *p* = 0.016); spirituality (-0.421 SMD, *p* < 0.001); and forgiveness (-0.200 SMD, *p* = 0.013). Last, strong invariance was also established across study condition assignments in the community-based sample, with intervention-assigned youth reporting significantly lower levels of hopeful future expectations (-0.245 SMD, *p* = 0.008) compared to waitlist-control-assigned youth.

## Discussion

Programs seeking to promote the positive development of youth often take a strengths-based PYD approach, increasingly in the majority world (YouthPower Learning, 2017). PYD approaches have their foundation in contemporary models of human development that integrate individuals and contexts in mutually influential ways (e.g., relational-developmental systems metatheory; see Overton, 2015). Organizations such as USAID, UNICEF, and World Bank are increasingly aligning with such contemporary models of human development. The GROW Strong and GROW Free programs are examples of programs taking a PYD approach to promoting health and thriving in Zambia, seeking to address problems of unhealthy alcohol use.

To meaningfully assess the impact and effectiveness of the GROW programs as part of the broader *GROW Hopes for Life* study, in the present article, we reported results from our efforts using baseline, pre-intervention data to develop ecologically and psychometrically valid measures of character strengths specifically promoted by GROW—hope, forgiveness, spirituality, prudence, and self-control—that can be useful for future program evaluations. The researcher-practitioner partnership team, including members from Zambia and the U.S., compiled existing measures of these constructs and drafted additional items pertinent to the goals of the programs. A rigorous and iterative series of EFAs and CFAs was tested to refine the constructs for parsimony and robustness across demographic subgroups (see Duncan et al., 2014).

Notably, these preliminary tests resulted in different factor structures for the school-based (ages 13 to 17 years) and community-based (ages 18 to 24 years) samples: a 16-item, four-factor measure for the younger, school-based sample; and a 44-item, six-factor measure for the older, community-based sample. Following the orthogenetic principle (Werner, 1948, 1957)—which states that development proceeds from a state of relative globality and lack of differentiation, to states of increasing differentiation, integration, and hierarchic organization—it might be the case that these differences in factor structures are reflecting developmental differences across age, with relative globality of items and constructs reflected in the younger sample, and increasing differentiation occurring among the older sample. As found by Shubert et al. (2019), evidence exists that the latent structure of character strengths differentiate across childhood and adolescence, consistent with the orthogenetic principle of development.

For instance, for the younger youth, a good-fitting factor for spirituality could not be established. The lack of a distinct spirituality factor may be due to a ceiling effect and lack of variance in responses; or it may be due to the orthogenetic principle (Werner, 1948, 1957), such that the positive spirituality-related items were not differentiated enough from the other items, which may be expected among younger youth. In turn, for older youth, a meaningful spirituality factor was demonstrated. In addition, for younger youth, prudence and self-control were not distinct at the latent level; but for older youth, a good-fitting model could be established with distinct factors, though the correlation between these latent factors was still high. Perhaps similar to Piagetian notions of assimilation and accommodation (e.g., Piaget, 1952, 1977; see also Wapner & Kaplan, 2024), it may be that developmental differences in cognition and language contribute to differential understandings or interpretations of words and items intended to reflect these factors, further explaining the increasing differentiation that is described in the orthogenetic principle. Further tests of the ontology of character strengths would be needed to assess whether the orthogenetic principle is a helpful interpretation for these differences.

We then sought to establish between-group measurement invariance across subgroups present in the data set. Establishing strong measurement invariance provides justification for the use of these measures and for the interpretation of findings when comparing groups (Little, 1997). This analytic step was important as many measures—particularly those developed in the minority world—do not necessarily possess the psychometric qualities needed to assess youth in the majority world when translation procedures alone are enacted (e.g., see YouthPower Learning, 2017).

In the school-based sample, invariance tests demonstrated that the measures could justifiably be used to make comparisons across genders as well as across study conditions. These tests also revealed that youth assigned to the intervention group reported significantly lower levels of hope through hardship and of prudence/self-control compared to waitlist control youth; subsequent analyses comparing these groups will therefore have to consider these baseline differences. Invariance was also demonstrated across older and younger subsets of youth; however, model fit was not acceptable, suggesting that additional refinements to measures would be needed if trying to draw age-based conclusions using these measures in the school-based sample. Invariance was not established across urban and rural locations of participants, suggesting that urban youth and rural youth might interpret these items differently and thus comparisons should be made with caution as meaningful interpretation of findings might not be empirically justified. Future studies might seek to establish partial invariance across urban/rural locations to further illuminate those possible differences. Taken together, for the school sample, the measures established will be useful for assessing two types of hope (hope through hardship and hopeful future expectations), forgiveness, and a global prudence/self-control factor, and to make comparisons on those factors across the study conditions and across gender.

In the community-based sample, invariance tests supported the use of the measures to compare across genders, age, urban/rural location, and study condition. However, model fit was questionable across urban/rural groups, so interpretations should be made with caution and future studies might consider further refining the models to achieve acceptable fit. In regard to baseline differences, community-based young adults reported significantly lower levels of hopeful future expectations and of spirituality compared to younger community-based youth. Furthermore, rural youth reported significantly lower levels of all character strengths compared to urban youth. Reasons explaining these differences in self-appraisals require further research, but may be due to developmental differences in regard to age (e.g., older youth having a more differentiated and thus self-critical view of the items compared to younger youth) and culture (e.g., rural youth perhaps having different cultural experiences and perceptions of character strengths compared to urban youth) (e.g., Bornstein, 2017, 2019; Werner, 1948, 1957). Differences in hope and spirituality may also be related to heavy alcohol use (e.g., see Tumwesigye et al., 2013 in Uganda), which the broader *GROW Hopes for Life* study aims to assess in Zambia using measure validated in the present study. Last, regarding study condition, youth assigned to the intervention group reported significantly lower levels of hopeful future expectations, which should be considered in subsequent analyses. Taken together, for the Hopes for Life program, these tests resulted in robust measures that will be useful for assessing character strengths across groups present in the study.

### Limitations and Future Directions

These findings present promising first steps in establishing robust and psychometrically strong measures for assessing character strengths among youth in Zambia and perhaps in other Sub-Saharan African countries. At the same time, these results are preliminary and should be considered in light of their limitations. The data tested were cross-sectional and the relations assessed do not indicate usefulness of these measures for assessing trajectories of change, which the GROW programs hope to promote. Therefore, longitudinal data are needed to better understand the usefulness of these measures in assessing character development, particularly as a result of program engagement. Future studies from the *GROW Hopes for Life* study will seek to contribute such progress.

Limitations also pertain to sampling in the present study. The data sets are focused on the Zambian context and, although establishing ecologically valid (context-specific) measures for use in the present study, it was not possible to assess the diversity of life experiences that might contribute to baseline differences found at the latent levels of the constructs assessed. Regarding the school sample, these school-based youth may differ from youth who were not enrolled in school, perhaps due to lower socioeconomic status, lack of parental involvement, a history of lower school performance, or other factors. Community participants ages 18–24 may differ from peers who are not using alcohol in an unhealthy manner. Future studies should not only seek to validate these measures in additional diverse samples but, as well, should seek to move toward fully person-specific assessments of character development.

Additional limitations pertain to the self-report nature of the measures established. Self-report measures are generally skewed due to common-method variance, response-style bias, and social-desirability bias. Future research should triangulate findings with other data, for instance, from peer- and parent/guardian-report and qualitative interviews, to provide richer data for analysis and illumination of findings.

### Conclusions

This preliminary study demonstrates the development and refinement of meaningful and useful measures for assessing character strengths in the Zambian context. As part of the broader *GROW Hopes for Life* study, the measure-development steps documented here provide the statistical justification for using these measures and comparing groups in subsequent evaluations of the GROW Hopes for life programs. Given the need to document and evaluate PYD programs being implemented, particularly programs in the majority world and programs with a theoretical basis and robust measurement of PYD outcomes (see YouthPower Learning, 2017), these findings are timely and important. Scientists and society have the opportunity and the means to support and promote youth thriving. With the focus of the GROW programs, this study represents an initial step toward documenting and evaluating the effectiveness of offering culturally adapted training programs that target the development of specific character strengths for the purpose of lowering maladaptive behaviors and promoting adaptive behaviors that contribute more broadly to PYD.

## Supplementary information

Below is the link to the electronic supplementary material.

Supplementary file1 (DOCX 43 KB)

## Declarations

### Use of Original Data

The authors take responsibility for the integrity of the data and the accuracy of the data analyses.
