# Supplementary material for: Measuring Character Strengths and Promoting Positive Youth Development in Zambia: Initial Findings from the GROW Hopes for Life Study
Source: Child Youth Care Forum. 2024 Aug 1;54(2):303–22. doi: 10.1007/s10566-024-09814-8 (PMC11920339; doi:10.1007/s10566-024-09814-8)
Supplement: Supplementary file 1 — Supplementary file1 (DOCX 43 KB) [file 10566_2024_9814_MOESM1_ESM.docx]

Supplementary Table 1

| *All Character Strengths Items Tested for the Constructs in the Present Study* | | |
| --- | --- | --- |
| Construct | Item (*Stem*) | Source |
| Hope | *For each of the following questions, please choose a number from 0 to 10 for your answer, with 0 meaning you “Completely disagree” with that statement to 10 meaning you “Completely agree” with that statement.* |  |
|  | 1. I believe that things will always work out no matter how difficult they seem now. | VIA-IS |
|  | 1. I will achieve my goals. | VIA-IS |
|  | 1. Even when things look bad, I stay hopeful. | VIA-IS |
|  | 1. I know I can get through bad times. | VIA-IS |
|  | 1. I can live a good life without using alcohol. | Present study |
|  | 1. When I set goals, I can begin to accomplish my dreams. | Present study |
|  | *The next set of questions ask about things that could happen in your future. Read each sentence and choose a number from 0 to 10, with 0 meaning you think there is a 0% chance that this will happen in your future (“It will never happen”) to 10 meaning you think there is a 100% chance that this will happen in your future (“You are completely certain”).* |  |
|  | 1. Graduating high school. | HFE |
|  | 1. Going to college. | HFE |
|  | 1. Have a job that pays well. | HFE |
|  | 1. Having a job that you like. | HFE |
|  | 1. Having a happy family life. | HFE |
|  | 1. To live wherever I want. | HFE |
|  | 1. To buy what I need. | HFE |
|  | 1. To do the things I'd like to. | HFE |
|  | 1. Be respected in my community. | HFE |
|  | 1. Have trustworthy friendships. | HFE |
|  | 1. Being healthy. | HFE |
|  | 1. Being safe. | HFE |
| Forgiveness | *Think about when someone has hurt you or upset you. From 0, “Completely disagree,” to 10, “Completely agree,” much do you agree or disagree with the following statements about how you would respond?* |  |
|  | 1. I let go of my bad feelings. | CI Study of PYD |
|  | 1. I focus on the good in them. | CI Study of PYD |
|  | 1. I forgive them. | CI Study of PYD |
|  | 1. I can still be friends with people who have been mean to me. | Present study |
|  | 1. When someone says they're sorry for hurting me, I forgive them. | Present study |
|  | 1. When someone apologizes, I give them a second chance. | Present study |
|  | *From 0, “Completely disagree,” to 10, “Completely agree,” how much do you agree or disagree with each of the following statements?* |  |
|  | 1. I have forgiven myself for things that I have done wrong. | BMMRS |
|  | 1. I have forgiven those who hurt me. | BMMRS |
|  | 1. I know that God forgives me. | BMMRS |
|  | 1. I am a forgiving person. | VIA-IS |
| Spirituality | *On a scale from 0, “Completely disagree,” to 10, “Completely agree,” how much do you agree or disagree with each of the following statements?* |  |
|  | 1. I have faith in a "Higher Power" or God. | VIA-IS |
|  | 1. I believe all things happen for a reason. | VIA-IS |
|  | 1. I pray to a "Higher Power" or God. | VIA-IS |
|  | 1. I feel connected with God. | MDAS |
|  | 1. Connecting with God helps me find purpose in my life. | MDAS |
|  | 1. I am amazed at nature and God's creation. | MDAS |
|  | 1. I feel God's presence in my life. | MDAS |
|  | 1. I believe that God cares about me. | MDAS |
| Prudence | *On a scale from 0, “Completely disagree,” to 10, “Completely agree,” how much do you agree or disagree with each of the following statements?* |  |
|  | 1. I am very careful at whatever I do. | VIA-IS |
|  | 1. I think carefully before I act. | VIA-IS |
|  | 1. I think about the consequences of my behavior before I take action. | VIA-IS |
|  | 1. I rarely do bad things. | VIA-IS |
|  | 1. I try not to do anything that I will regret later. | Present study |
|  | 1. I plan before I make a major change. | Present study |
|  | 1. I pause to think before I make a major decision. | Present study |
| Self-control | *On a scale from 0, “Completely disagree,” to 10, “Completely agree,” how much do you agree or disagree with the following statements?* |  |
|  | 1. If I want something, I can wait for it. | VIA-IS |
|  | 1. I am able to control my anger well. | VIA-IS |
|  | 1. I have a lot of patience. | VIA-IS |
|  | 1. I rarely lose my temper. | VIA-IS |
|  | 1. Even when I am under pressure, I can control my emotions. | Present study |
|  | 1. Even when other are drinking alcohol, I am able to say no. | Present study |
|  | 1. Even when no one is watching, I try to do the right thing. | Present study |
| *Note:* VIA-IS = VIA Inventory of Strengths (Park & Petersen, 2006); HFE = Hopeful Future Expectations scale (Schmid et al., 2011); CI Study of PYD = Compassion International Study of Positive Youth Development (e.g., Tirrell et al., 2019, 2020, 2022); BMMRS = Brief Multidimensional Measure of Religiousness/Spirituality (Masters, 2020); MDAS = Measure of Diverse Adolescent Spirituality (King et al., 2017; see also Tirrell et al., 2019); Present study refers to items generated by the research team to test in the GROW Hopes for Life study. | | |

Supplementary Table 2

| *Final Items and Constructs Validated with the School-Based Sample in the Present Study* | | |
| --- | --- | --- |
| Construct | Item (*Stem*) | Source |
| Hope (through hard times) | *For each of the following questions, please choose a number from 0 to 10 for your answer, with 0 meaning you “Completely disagree” with that statement to 10 meaning you “Completely agree” with that statement.* |  |
|  | 1. I believe that things will always work out no matter how difficult they seem now. | VIA-IS |
|  | 1. Even when things look bad, I stay hopeful. | VIA-IS |
|  | 1. I know I can get through bad times. | VIA-IS |
| Hope (hopeful future expectations) | *The next set of questions ask about things that could happen in your future. Read each sentence and choose a number from 0 to 10, with 0 meaning you think there is a 0% chance that this will happen in your future (“It will never happen”) to 10 meaning you think there is a 100% chance that this will happen in your future (“You are completely certain”).* |  |
|  | 1. Having a happy family life. | HFE |
|  | 1. To live wherever I want. | HFE |
|  | 1. To buy what I need. | HFE |
|  | 1. Being healthy. | HFE |
|  | 1. Being safe. | HFE |
| Forgiveness | *Think about when someone has hurt you or upset you. From 0, “Completely disagree,” to 10, “Completely agree,” much do you agree or disagree with the following statements about how you would respond?* |  |
|  | 1. I let go of my bad feelings. | CI Study of PYD |
|  | 1. I focus on the good in them. | CI Study of PYD |
|  | 1. I forgive them. | CI Study of PYD |
|  | 1. I can still be friends with people who have been mean to me. | Present study |
| Prudence | *On a scale from 0, “Completely disagree,” to 10, “Completely agree,” how much do you agree or disagree with each of the following statements?* |  |
|  | 1. I am very careful at whatever I do. | VIA-IS |
|  | 1. I think carefully before I act. | VIA-IS |
|  | 1. I plan before I make a major change. | Present study |
|  | 1. Even when I am under pressure, I can control my emotions. | Present study |
| *Note:* A good-fitting model could not be established with the *spirituality* items for the school-based sample, perhaps due to a ceiling effect of high mean scores and low variance for the items. Items for *prudence* and *self-control* did not form a distinct latent factors in the school-based sample. VIA-IS = VIA Inventory of Strengths (Park & Petersen, 2006); HFE = Hopeful Future Expectations scale (Schmid et al., 2011); CI Study of PYD = Compassion International Study of Positive Youth Development (e.g., Tirrell et al., 2019, 2020, 2022); BMMRS = Brief Multidimensional Measure of Religiousness/Spirituality (Masters, 2020); MDAS = Measure of Diverse Adolescent Spirituality (King et al., 2017; see also Tirrell et al., 2019); Present study refers to items generated by the research team to test in the GROW Hopes for Life study. | | |

Supplementary Table 3

| *Final Items and Constructs Validated with the Community-Based Sample in the Present Study* | | |
| --- | --- | --- |
| Construct | Item (*Stem*) | Source |
| Hope (through hard times) | *For each of the following questions, please choose a number from 0 to 10 for your answer, with 0 meaning you “Completely disagree” with that statement to 10 meaning you “Completely agree” with that statement.* |  |
|  | 1. I believe that things will always work out no matter how difficult they seem now. | VIA-IS |
|  | 1. I will achieve my goals. | VIA-IS |
|  | 1. Even when things look bad, I stay hopeful. | VIA-IS |
|  | 1. I know I can get through bad times. | VIA-IS |
|  | 1. I can live a good life without using alcohol. | Present study |
|  | 1. When I set goals, I can begin to accomplish my dreams. | Present study |
| Hope (hopeful future expectations) | *The next set of questions ask about things that could happen in your future. Read each sentence and choose a number from 0 to 10, with 0 meaning you think there is a 0% chance that this will happen in your future (“It will never happen”) to 10 meaning you think there is a 100% chance that this will happen in your future (“You are completely certain”).* |  |
|  | 1. Have a job that pays well. | HFE |
|  | 1. Having a job that you like. | HFE |
|  | 1. Having a happy family life. | HFE |
|  | 1. To live wherever I want. | HFE |
|  | 1. To buy what I need. | HFE |
|  | 1. To do the things I'd like to. | HFE |
|  | 1. Be respected in my community. | HFE |
|  | 1. Have trustworthy friendships. | HFE |
|  | 1. Being healthy. | HFE |
|  | 1. Being safe. | HFE |
| Forgiveness | *Think about when someone has hurt you or upset you. From 0, “Completely disagree,” to 10, “Completely agree,” much do you agree or disagree with the following statements about how you would respond?* |  |
|  | 1. I focus on the good in them. | CI Study of PYD |
|  | 1. I forgive them. | CI Study of PYD |
|  | 1. I can still be friends with people who have been mean to me. | Present study |
|  | 1. When someone says they're sorry for hurting me, I forgive them. | Present study |
|  | 1. When someone apologizes, I give them a second chance. | Present study |
|  | *From 0, “Completely disagree,” to 10, “Completely agree,” how much do you agree or disagree with each of the following statements?* |  |
|  | 1. I have forgiven myself for things that I have done wrong. | BMMRS |
|  | 1. I have forgiven those who hurt me. | BMMRS |
|  | 1. I am a forgiving person. | VIA-IS |
| Spirituality | *On a scale from 0, “Completely disagree,” to 10, “Completely agree,” how much do you agree or disagree with each of the following statements?* |  |
|  | 1. I have faith in a "Higher Power" or God. | VIA-IS |
|  | 1. I believe all things happen for a reason. | VIA-IS |
|  | 1. I pray to a "Higher Power" or God. | VIA-IS |
|  | 1. I feel connected with God. | MDAS |
|  | 1. Connecting with God helps me find purpose in my life. | MDAS |
|  | 1. I am amazed at nature and God's creation. | MDAS |
|  | 1. I feel God's presence in my life. | MDAS |
|  | 1. I believe that God cares about me. | MDAS |
| Prudence | *On a scale from 0, “Completely disagree,” to 10, “Completely agree,” how much do you agree or disagree with each of the following statements?* |  |
|  | 1. I am very careful at whatever I do. | VIA-IS |
|  | 1. I think carefully before I act. | VIA-IS |
|  | 1. I think about the consequences of my behavior before I take action. | VIA-IS |
|  | 1. I try not to do anything that I will regret later. | Present study |
|  | 1. I plan before I make a major change. | Present study |
|  | 1. I pause to think before I make a major decision. | Present study |
| Self-control | *On a scale from 0, “Completely disagree,” to 10, “Completely agree,” how much do you agree or disagree with the following statements?* |  |
|  | 1. If I want something, I can wait for it. | VIA-IS |
|  | 1. I am able to control my anger well. | VIA-IS |
|  | 1. I have a lot of patience. | VIA-IS |
|  | 1. I rarely lose my temper. | VIA-IS |
|  | 1. Even when I am under pressure, I can control my emotions. | Present study |
|  | 1. Even when no one is watching, I try to do the right thing. | Present study |
| *Note:* VIA-IS = VIA Inventory of Strengths (Park & Petersen, 2006); HFE = Hopeful Future Expectations scale (Schmid et al., 2011); CI Study of PYD = Compassion International Study of Positive Youth Development (e.g., Tirrell et al., 2019, 2020, 2022); BMMRS = Brief Multidimensional Measure of Religiousness/Spirituality (Masters, 2020); MDAS = Measure of Diverse Adolescent Spirituality (King et al., 2017; see also Tirrell et al., 2019); Present study refers to items generated by the research team to test in the GROW Hopes for Life study. | | |
